# Portage de l'antigène HBs et des anticorps anti-VHC chez le drépanocytaire homozygote à l'Hôpital Central de Yaoundé

**DOI:** 10.11604/pamj.2013.14.40.2069

**Published:** 2013-01-28

**Authors:** Françoise Ngo Sack, Dominique Noah Noah, Haman Zouhaïratou, Dora Mbanya

**Affiliations:** 1 Faculté de médecine et des sciences pharmaceutiques de l'université de Douala, Douala, Cameroun; 2 Hôpital central de Yaoundé, Yaoundé, Cameroun; 3 Faculté de médecine et des sciences biomédicales de l'université de Yaoundé 1, Yaoundé, Cameroun; 4 Centre hospitalier et universitaire de Yaoundé, Yaoundé, Cameroun

**Keywords:** Prévalence, Antigène HBs, anticorps Anti-HCV, drépanocytose, Yaoundé, Prevalence, Antigen HBs, antibody Anti-HCV, sickle cell, Yaoundé

## Abstract

**Introduction:**

La drépanocytose est un problème de santé publique en Afrique subsahérienne où le portage hétérozygote varie de 20 et 25%. Elle se manifeste essentiellement par des crises vaso-occlusives et/ou hémolytiques et nécessite souvent une thérapeutique transfusionnelle, nonobstant un risque demeuré élevé dans les pays en développement. Le but de cette étude était d’évaluer le portage de l'Ag HBs et de l'Ac anti HCV chez les drépanocytaires homozygotes à l'hôpital central de Yaoundé (HCY).

**Méthodes:**

Pendant une période allant d'août 2008 à janvier 2009, nous avons recruté les patients drépanocytaires régulièrement suivi dans le service d'hématologie de l'HCY. Pour chaque individu, un prélèvement de 5 ml de sang veineux a été effectué. Le sérum était conservé à -20°C jusqu’à l'utilisation pour le dépistage de l'antigène HBs et des anticorps anti-VHC.

**Résultats:**

108 patients drépanocytaires homozygotes ont participé à cette étude. Selon le sexe, 57 soit 52,80% étaient de sexe masculin et 51 soit 47,20% de sexe féminin. Les patients étaient âgés de 5 à 47 ans avec un âge moyen de 21,45 ± 9 ans. Sept (7) patients soit 6,48% étaient positif pour l'Ag HBs, 18 patients soit 16,67% étaient positifs pour l'Ac anti-HCV. Le nombre de patient ayant reçu au moins une transfusion antérieurement était de 93 soit 86.1%. Le nombre de patients ayant reçu plus de 10 transfusions était de 14 soit 13%. Nous avons une prévalence de 42,86% de positivité de l'Ac anti-HCV pour la population drépanocytaire ayant reçu plus de 10 transfusions contre 12,77% pour celle ayant reçu moins de 10 transfusions (P < 0,01).

**Conclusion:**

La prévalence de l'antigène HBs, de l'Ac anti-VHC chez les drépanocytaires homozygotes à Yaoundé est élevée. Cette prévalence croît avec le nombre de transfusions reçues, surtout chez les patients ayant reçu plus de 10 transfusions sanguines. Ces résultats posent le problème de la sécurité transfusionnelle qui doit utiliser les méthodes modernes comme dans les pays développés où le risque résiduel de transfusion sanguine est quasi nul. Les politiques sanitaires des pays africains subsahariens dont le Cameroun, doivent systématiser la vaccination contre l'HVB chez toutes les personnes à risque dont les drépanocytaires.

## Introduction

La drépanocytose est un problème de santé publique en Afrique subsahérienne où le portage hétérozygote varie de 20 et 25%. D'après l'organisation Mondiale de la Santé (OMS), plus de 240 millions de patients présentent une hémoglobinopathie hétérozygote et chaque année il nait au moins 200 000 enfants atteints d'hémoglobinopathie homozygote létale se répartissant à peu près par moitié entre la thalassémie et la drépanocytose [1]. Cette maladie qui se manifeste essentiellement par des crises vaso-occlusives et/ou hémolytiques nécessite souvent une thérapeutique transfusionnelle. En effet, la transfusion sanguine est une arme essentielle de la prise en charge des complications graves de la drépanocytose. Mais ses indications et ses modalités sont aujourd'hui réévaluées en fonction des nouvelles thérapeutiques de la drépanocytose actuellement disponibles telle que l'hydroxyurée ou mieux maîtrisées telle que allogreffe de moelle osseuse, de la nécessité d'une sécurité transfusionnelle maximale. Dans certaines situations d'urgence, la transfusion est le seul traitement efficace pour améliorer l'oxygénation tissulaire et limiter la gravité du phénomène vaso-occlusif en épurant l'hémoglobine S. Cette transfusion peut être simple dans les cas d'anémie profonde ou d'hypovolémie aiguë. On peut aussi procéder à des échanges transfusionnels ponctuels dans certaines complications aiguës vaso-occlusives ou infectieuses [2].

On estime à 350 millions le nombre de porteurs asymptomatiques du VHB dans le monde, à 100 millions le nombre de cirrhoses hépatiques et à 2 millions le nombre de décès annuels par cirrhose ou cancer du foie [3]. La maladie est endémique en Afrique, au sud du Sahara, dans le bassin de l'Amazonie à Haïti et en République Dominicaine. Dans ces régions, environ 20% de la population sont infectés dans l'enfance. La séroprévalence varie entre 3 et 15% [4]. Les infections par le VHC sévissent dans le monde entier. La proportion des sujets infectés pourrait atteindre 3% de la population mondiale et il pourrait y avoir plus de 170 millions de porteurs chroniques exposés aux risques de cirrhose et à l'hépatocarcinome. La prévalence est estimée à 1,7% en Amérique, 1,9% en Europe, 2,15% en Asie du Sud-est, 4,6% dans le pourtour méditerranéen et 5,3% en Afrique [4]. Au Cameroun en 1995, Kowo et al retrouvent dans les populations pygmée et bantou une prévalence de l'hépatite C estimée à 13% [5].

Une étude menée à l'hôpital Central de Yaoundé en 2009 a montré que La transmission parentérale du virus de l'Hépatite B (VHB) lors des transfusions sanguines n'est pas négligeable. Même si la sécurité transfusionnelle s'est beaucoup renforcée au cours de ces 15 dernières années, le risque transfusionnel demeure élevé dans les pays en développement. Dans le contexte de sécurité transfusionnelle limitée dans certains hôpitaux au Cameroun, la mise en place des bonnes pratiques de qualification des dons sanguins basées sur l'utilisation des techniques plus sensibles pour le dépistage du risque infectieux des dons de sang devrait constituer une priorité des autorités sanitaires [6]. L'objectif général de cette étude est d’évaluer le portage de l'Ag HBs et de l'Ac anti HCV chez les drépanocytaires homozygotes à l'hôpital central de Yaoundé.

## Méthodes

Pendant une période de six mois allant d'août 2008 à janvier 2009, nous avons mené cette étude prospective, descriptive, transversale et analytique, dans le service d'hématologie et d'oncologie médicale de l'Hôpital Central de Yaoundé (HCY) qui accueille surtout les adultes. Les enfants de moins de cinq ans étaient pour la plupart dirigés à la Fondation Chantal Biya (FCB) qui est un hôpital pédiatrique.

Après consentement éclairé, tout drépanocytaire homozygote connu vivant à Yaoundé et régulièrement suivi dans le service était inclus dans l’étude. Pour chaque individu, un prélèvement de 5 ml de sang veineux périphérique a été effectué dans un tube sec de type Vacutainer. Le sérum était recueilli dans l'heure qui suivait et conservé à -20°C jusqu’à l'utilisation pour le dépistage de l'antigène HBs et des anticorps anti-VHC. Les techniques de dépistage rapide Orea HBsAg^®^ et Orea HCV^®^ (Laboratoires Biocentric, France) étaient utilisées pour la détection qualitative de la présence de l'Ag HBs et de l'Ac anti-VHC. La confirmation de la positivité était effectuée en utilisant le réactif Elisa Fortress^®^ (Fortress Diagnostics, Royaume-Uni).

Ce travail a été approuvé par le comité éthique de l'Hôpital Central de Yaoundé. L'analyse statistique des données a été réalisée à l'aide des logiciels Excel, Spss 10.1 et Epi Info 2007 pour Windows. L'analyse descriptive a été réalisée grâce aux calculs des proportions pour les variables qualitatives (fréquence, pourcentage), des moyennes et des écarts types pour les variables continues. Les différentes comparaisons de fréquence ont été faites à l'aide du test Chi-carré (X^2^) de Pearson et le test de Fisher si nécessaire.

## Résultats

**Figure 1:**
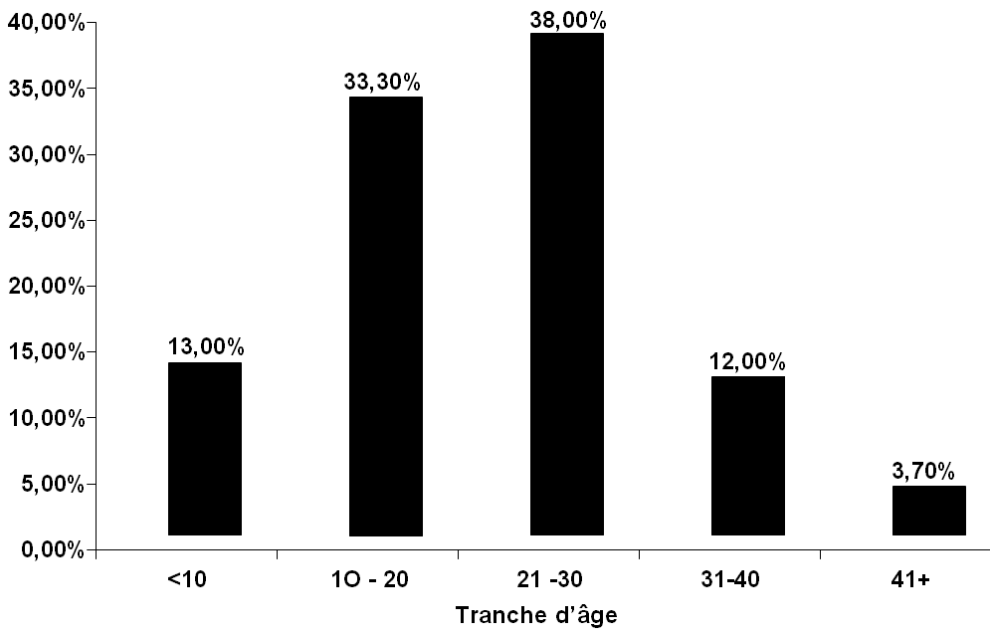
Répartition de la population d’étude selon l’âge

**Figure 2:**
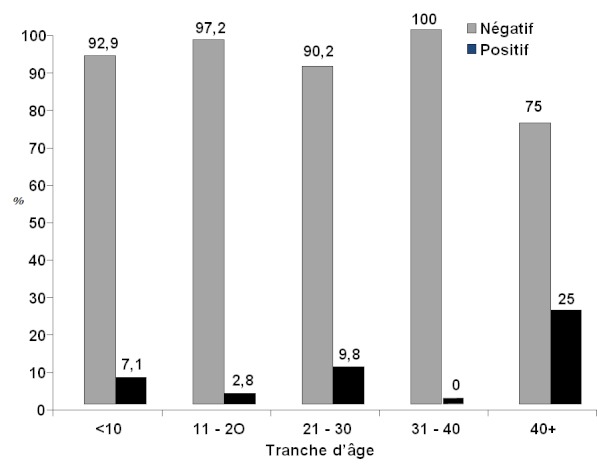
Prévalence de l'Ag HBs en fonction de l’âge

Au total 108 patients drépanocytaires homozygotes ont participé à cette étude. Selon le sexe, 57 soit 52,80% étaient de sexe masculin et 51 soit 47,20% de sexe féminin. Le sex ratio homme/femme était de 1,12. Les patients étaient âgés de 5 à 47 ans avec un âge moyen de 21,45 ± 9 ans. Selon les tranches d’âge on notait: tranche d’âge de moins de 10 ans: 14 patients soit 13%; tranche d’âge de 10 à 20 ans: 36 patients soit 33,3%; tranche d’âge de 21 à 30 ans: 41 patients soit 38%; tranche d’âge de 31 à 40 ans: 13 patients soit 12%; tranche d’âge de plus de 40 ans: 4 patients soit 3,7% (Figure 1) Sept (7) patients soit 6,48% étaient positif pour l'Ag HBs (Figure 2), 18 patients soit 16,67% étaient positifs pour l'Ac anti-HCV. Le nombre de patient ayant reçu au moins une transfusion antérieurement était de 93 soit 86.1%. Le nombre de patients ayant reçu plus de 10 transfusions était de 14 soit 13%.

Sur les 7 patients positifs à l'Ag HBs, 6 (85,70%) avaient déjà reçu au moins une transfusion antérieurement. Concernant la séropositivité à l'Ac anti-HCV, 17 (94,44%) patients sur 18 avaient été transfusés au moins une fois (P = 0,015). Nous avons une prévalence de 42,86% (6 sur 14) de positivité de l'Ac anti-HCV pour la population drépanocytaire ayant reçu plus de 10 transfusions contre 12,77% pour celle ayant reçu moins de 10 transfusions (P < 0,01).

## Discussion

Le nombre de patient ayant participé à notre étude était de 108 avec un sex ratio de 1,12 en faveur des hommes. Plusieurs études ont montré la prédominance du sexe masculin dans la prévalence des hépatites d'une manière générale. C. Mbendi Nlombi et al. dans une étude réalisée dans trois hôpitaux de Kinshasa-Est en RDC ont montré que la prévalence de l'Ag HBS chez les donneurs de sang est prédominante chez les hommes [7].

Une enquête de l'Institut de Veille Sanitaire réalisée en 2003-2004 en France a montré que les personnes porteuses chroniques du VHB étaient majoritairement de sexe masculin (les hommes sont 7 fois plus représentés que les femmes), en situation de précarité sociale (3 fois plus de bénéficiaires de la Caisse Mutuelle Universelle) (CMUc) et nées notamment en Afrique sub-saharienne (5 fois plus que celles nées en Europe), au Moyen-Orient (2,5 fois plus) et en Asie (2 fois plus) [8].

Les prévalences globales retrouvées ici étaient de 6,48% pour l'Ag HBs, et 16,67% pour l'Ac anti-HCV. Ces prévalences se rapprochent de celle obtenue par Dokekias et al. en 2001 où la prévalence de l'anticorps anti-HCV chez les malades drépanocytaires à Brazzaville était de 16,9% [9]. Kodjoh et al. en 1996 au cours d'une étude de la prévalence de l'hépatite C chez les drépanocytaires à Cotonou au Benin, retrouvent une prévalence de 20% [10]. Au Cameroun en 1996, Kemmegne et al. avaient eu une prévalence du HCV chez les drépanocytaires estimée à 23% [11]. Cette différence pourrait s'expliquer par le fait que cette prévalence était obtenue uniquement chez les drépanocytaires qui avaient été polytransfusés et donc plus à risque d’être infectés. On peut souligner pour le déplorer le rôle de la transmission sanguine dans nos pays africains qui n'a pas encore atteint le stade sécuritaire tel qu'il existe actuellement dans les pays développés. En effet, le dépistage des hépatites B et C ne faisait pas partie au Cameroun des examens de routine pratiqués chez les donneurs de sang avant l'année 2005. Par ailleurs, une étude réalisée par Noah Noah et al. chez les donneurs de sang à l'hôpital central de Yaoundé a montré que le risque résiduel infectieux restait élevé (9,8%) avec les méthodes utilisées [6]. En tenant compte du fait que de nombreux patients ont été transfusés avant l'instauration de ces mesures, on comprend pourquoi les prévalences globales des hépatites B et C chez les drépanocytaires polytransfusés sont élevées.

La tranche d’âge de 21-30 ans avec 4 patients positifs sur 7 à l'Ag HBs est celle qui est la plus touchée et c'est également dans cette tranche d’âge que les patients sont les plus sexuellement actifs, argument qui ne peut être négligé pour l'antigène HBs. Par ailleurs, d'autres éléments peuvent expliquer que cette tranche d’âge soit la plus concernée; le virus de l'hépatite B est connu comme 100 fois plus contagieux que le VIH, l'usage des drogues intraveineuse bien que peu répandu en Afrique concerne aussi cette tranche d’âge, l'HVB étant une maladie silencieuse, c'est également dans cette tranche d’âge qu'apparaissent généralement les complications. On note cependant qu'il n'y a aucune corrélation statistiquement significative (P = 0,988) entre le nombre d'unités de sang reçues et la séropositivité à l'Ag HBs.

Globalement, la prévalence de l'Ac anti-HCV augmente avec l’âge, la majorité étant entre 31 et 47 ans (Figure 2). Ceci était confirmé par l’étude de Courouce et al. en 1998; les données de l'Agence Française du Sang selon lui, soulignaient que la prévalence de l'hépatite C croît avec l’âge en raison de multiples modes de contamination [12]. La séropositivité à l'Ac anti-HCV augmente aussi dans la population transfusée en fonction du nombre de transfusions reçues (Tableau 1), ce résultat était déjà obtenu par Kemmegne et al. en 1996 [11]. La voie transfusionnelle représente donc le mode de transmission par excellence de l'hépatite C et les résultats de Mrabet et al. en 1999 l'avaient montré lors d'une étude réalisée chez les donneurs de sang au Centre de Transfusion des Forces Armées Royales à Rabat [13].

**Tableau 1:** Répartition de la population selon le nombre de transfusions sanguines reçues

Nombre de transfusions	Fréquence	Pourcentage
0	15	13,9%
1-5	58	53,7%
6-10	21	19,4%
Plus de10	14	13,0%
**Total**	**108**	**100,0%**

**Figure 3:**
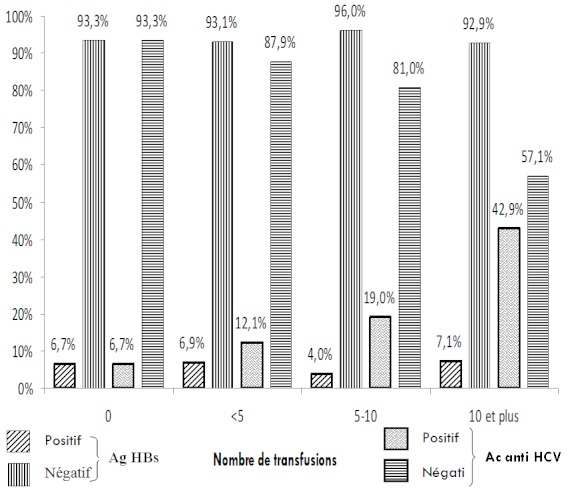
Prévalence de l'Ag HBs et l'Ac anti HCV selon le nombre de transfusions sanguines reçues

Nous avons dans notre étude des résultats qui montrent une différence, dans la population drépanocytaire ayant reçu plus de 10 unités de sang qui a une prévalence de 42,86%, contre 12,77% pour ceux ayant reçu moins de 10 unités (Figure 3). Devault et al. en 1994 rapportent, chez les 121 drépanocytaires ayant reçu plus de 10 unités de produits sanguins à Philadelphie (Pennsylvanie), une séroprévalence de l'hépatite C estimée à 20,7% et ce taux passe à 8,6% chez les sujets ayant reçu moins de 10 unités [14]. Ndumbe et al. relèvent lors d'une étude en 1993, une forte prévalence de l'infection à VHC chez l'enfant drépanocytaire au delà de 4 ans avec un taux de 31% [15]. L'importante prévalence des anticorps anti-HCV chez les patients drépanocytaires souvent très jeunes traduit surtout le risque transfusionnel majeur et probablement pas d'autres modes de contamination. Au Nigéria, dans une étude réalisée chez les donneurs de sang rémunérés et les patients drépanocytaires, la séroprévalence de l'hépatite C décrite par Mutimer et al. se situe à 14% [16].

## Conclusion

La prévalence de l'antigène HBs, de l'Ac anti-VHC chez les drépanocytaires homozygotes à Yaoundé est élevée. Cette prévalence croît avec le nombre de transfusions reçues, surtout chez les patients ayant reçu plus de 10 transfusions sanguines. Ces résultats posent le problème de la sécurité transfusionnelle qui doit utiliser les méthodes modernes comme dans les pays développés où le risque résiduel de transfusion sanguine est quasi nul. Les politiques sanitaires des pays africains subsahariens dont le Cameroun, doivent systématiser la vaccination contre l'HVB chez toutes les personnes à risque dont les drépanocytaires.
